# Long COVID management: a mini review of current recommendations and underutilized modalities

**DOI:** 10.3389/fmed.2024.1430444

**Published:** 2024-06-14

**Authors:** Tiffany K. Dietz, Kirsten N. Brondstater

**Affiliations:** School of Health Professions, Shenandoah University, Winchester, VA, United States

**Keywords:** long COVID, chronic COVID-19, post-acute sequelae of SARS-CoV-2, post COVID, management, low-dose naltrexone, stellate ganglion block

## Abstract

Long COVID is a condition that develops in a subset of patients after COVID-19 infection comprising of symptoms of varying severity encompassing multiple organ systems. Currently, long COVID is without consensus on a formal definition, identifiable biomarkers, and validated treatment. Long COVID is expected to be a long-term chronic condition for a subset of patients and is associated with suffering and incapacity. There is an urgent need for clear management guidelines for the primary care provider, who is essential in bridging the gap with more specialized care to improve quality of life and functionality in their patients living with long COVID. The purpose of this mini review is to provide primary care providers with the latest highlights from existing literature regarding the most common long COVID symptoms and current management recommendations. This review also highlights the underutilized interventions of stellate ganglion blocks and low-dose naltrexone, both with well-established safety profiles demonstrated to improve quality of life and functionality for patients suffering with some symptoms of long COVID, and encourages prompt referral to interventional pain management.

## 1 Introduction

The recent Coronavirus disease 2019 (COVID-19) pandemic elicits many challenges in healthcare. Severe acute respiratory syndrome coronavirus 2 (SARS-CoV-2) causes COVID-19 infection (1, 2). SARS-CoV-2 utilizes angiotensin-converting-enzyme 2 (ACE2) to bind with spike protein (S-protein) to enter the cell (2). ACE2 is found in several body tissues such as the heart, lungs, kidneys, and gastrointestinal system (3). Most people infected by COVID-19 make a full recovery however, there are varying estimates from 0 to 93% of those infected developing a long-term condition comprised of often severe symptoms affecting multiple organ systems known as long COVID (1, 2, 4, 5). These estimate are highly varied based on several factors such as the definition of long COVID, settings such as hospital-based versus outpatient, reporting methods such as medical records versus self-report, and vaccination status (5, 6). In a subset of patients, long COVID is expected to be a chronic illness with high impacts to healthcare utilization, workforce and employment. The long COVID burden is estimated to be $2.6 trillion per year in the USA (7). The pandemic united the world in a global effort for rapid development and delivery of COVID vaccinations but the lack of sufficient data and recommendations for managing long COVID complications remain.

There are no current validated biomarkers or validated treatments for long COVID making management difficult for providers and frustrating for patients already living with long COVID. Due to the lack of sufficient data many recommendations for managing long COVID are based on expert opinion thus there is urgent need for more research. Patients living with long COVID experience varying severity of debility. Many current strategies include self-management and rehabilitation. Proposed treatments include: apheresis (8), nirmatrelvir/ritonavir (Paxlovid) (9), antihistamines such as loratadine (10), fexofenadine, and famotidine (11), anticoagulants such as apixaban (10) and antiplatelet agents such as aspirin and clopidogrel (12). Thrombolytics such as nattokinase, serrapeptase, lumbrokinase, and bromelain have also been proposed as potential treatments for long COVID (13).

Missed opportunities for intervention are imputable to the paucity of randomized controlled trials. Long COVID patients are more likely to utilize services from their primary care providers to seek further care for their new chronic disease. The aim of this mini review is to provide primary care providers the latest highlights from existing literature regarding current recommendations and feature two safe and underutilized interventions that may be helpful in improving functionality and quality of life for their patients already suffering with some symptoms of long COVID with an emphasis on prompt referral to interventional pain management.

## 2 Background

COVID-19 was declared a pandemic on March 11, 2020 and as of March 2024, the World Health Organization reports over 775 million cases of COVID-19 worldwide; 103 million cases are in the United States alone (14). The prevalence of long COVID may be underestimated due to the breadth and variability of symptoms (15). There is consistent evidence that women are more likely to report symptoms of long COVID and have a diagnosis compared to men (6, 16–18). An analysis of repeat cross-sectional data collected by US Census Bureau from June 2022 to June 2023 determined the highest prevalence of long COVID and prevalence of associated significant activity limitation was found in adults 35–44 years of age (19). This group encompasses the largest portion of the US workforce as the median age of the labor force is 41.8 (20).

Long COVID pathophysiology remains unclear. Current hypotheses underlying pathophysiology include: Post-viral immune dysregulation triggering multi-organ inflammation, reactivation of latent pathogens, autoimmunity, and formation of microclots (21, 22).

Long COVID is without specific biomarkers for detection and without validated effective treatments (4, 18, 23). Over 200 symptoms encompassing multiple organ systems have been reported and severity varies from mild and reversible to moderate or severe and persistent (18). Long COVID is expected to be a long-term chronic condition for a subset of patients that may last months to years and is associated with suffering and incapacity highlighting an urgent need for clearer management guidelines and interventions.

## 3 Long COVID definitions

Long COVID has also been referred to as post COVID conditions (PCC), post-acute sequelae of SARS-CoV-2 infection (PASC), and long-haul COVID (7, 24, 25). In 2021, long COVID condition was assigned an International Classification of Diseases, Tenth Revision (ICD-10) code, U90.9 (26).

There is not an established definition for long COVID, though sometimes it includes symptom duration or clusters of symptoms and may not always be straightforward (27, 28). Raveendran (28) proposes clinical and essential criteria to facilitate categorizing long COVID into four categories: confirmed, probable, possible, or doubtful. The National Institute for Health and Care Excellence (NICE) defines long COVID as “signs and symptoms that continue or develop after acute COVID-19” and “includes both ongoing symptomatic COVID-19 (from 4 to 12 weeks) and post-COVID-19 syndrome (12 weeks or more)” (29). The National Institutes of Health (NIH) Researching COVID to Enhance Recovery (RECOVER) Initiative program defines long COVID as “ongoing, relapsing, or new symptoms, or other health effects occurring after the acute phase of SARS-CoV-2 infection (i.e., present four or more weeks after acute infection)” (22).

A Delphi process conducted in partnership between the World Health Organization (WHO) Clinical Case Definition Working Group on Post-COVID-19 Condition and an international panel of patients, providers, researchers and WHO staff, provides the following definition for long COVID: A condition occurring in individuals with SARS-CoV-2 infection history at least 3 months post-acute infection with persistent symptoms of at least 2 months duration in which an alternative diagnosis cannot be obtained (30). The US Centers for Disease Control and Prevention (CDC), defines long COVID as new onset or persistent symptoms of at least 4 weeks’ duration from acute infection (31). Commonalities in the definition of long COVID is comprised of signs and symptoms occurring 3 months after acute COVID infection and persistent for at least 2 months. Differences in the timelines defining long COVID have significant effects on comparing research among these studies. Several studies note that long COVID symptoms change over time from predominantly respiratory towards neuropsychiatric symptoms.

## 4 Preliminary workup

Diagnosing long COVID is difficult with over 200 associated/related symptoms. Long COVID is not exclusively associated with severe acute COVID infections. Many studies focus on hospitalized COVID patients thus there is an underrepresentation of non-hospitalized COVID patients (32). The most commonly reported long COVID symptoms include: fatigue, dyspnea, cognitive impairment, myalgias and arthralgias, headache, cough, chest pain, smell and taste alterations (17, 26, 33, 34). No specific biomarkers exist to detect long COVID (4, 15, 18) although proposed biomarkers include: C-reactive protein (35), interferon gamma (36), interleukin-6 (35, 37), and tumor necrosis factor alpha (38). Antibodies do not facilitate the retrospective diagnosis of infection in patients with long COVID. Antibodies are only found in about 80% of patients that seroconvert and these levels decrease over time and are undetectable at 3 months (26). Most studies measured spike antibodies, which are no longer relevant post-vaccination. Anti-nucleocapsid protein antibodies also diminish over 6 months in 50% of patients hospitalized with COVID-19 (39).

Assessment of patients with high suspicion for long COVID should include a comprehensive evaluation with collection of a thorough history. Physical examination should include baseline vital signs and bloodwork such as complete blood count, electrolytes, creatinine, random blood glucose, hemoglobin A1c, thyroid panel, and an electrocardiogram (EKG) (40). Tools such as the World Health Organization (WHO) Post-COVID-19 Functional Status (PCFS) Scale and the Montreal Cognitive Assessment (MOCA) test may be helpful to assess for resolution of acute COVID symptoms, recurrence of symptoms, and/or development of new symptoms within the first year following acute COVID infection (41). After thorough work-up to exclude other alternative diagnoses and referrals to appropriate specialists when indicated, management comprises of symptom control and interventions for treatable characteristics.

## 5 Management

Current and recommended management strategies differ widely across the literature. Strategies consist primarily of supportive care or based on system involvement. Current care recommendations include multidisciplinary rehabilitation, self-management and self-pacing (42–44). Self-management approaches include appropriate rest, practicing good sleep hygiene, energy pacing methods, diet control, and distraction (41, 42, 45). Pacing strategies are aimed at coping with inconsistent and decreased energy levels by adapting or adjusting efforts to different activities (43). This may include energy conservation which integrates activity prioritization, task delegation, assistive device utilization, and alternating between activity and rest to complete daily activities, and activity pacing which incorporates activity goals and gradual increase of activity levels (43). Higher pacing adherence is associated with higher rates of recovery and improvement (43).

Described hereafter are management recommendations for the most common long COVID presentations that cause the most morbidity and disability. Management recommendations by symptom is presented in Table 1.

**Table 1:** Long COVID intervention recommendations by symptom.

	Self-management^a^	Physical exercise rehabilitation	Pulmonary rehabilitation	SGB	LDN
Cardiopulmonary symptoms					
Fatigue	X	X^b^		X	X
Dyspnea			X	X	
CVD: Chest Pain, Palpitations, Fatigue, Brain Fog		X^b^		X	X
Neuropsychiatric symptoms					
Depression		X	X	X	
Anxiety		X	X	X	
Sleep disturbances	X	X		X	X
Parosmia				X	
Cognitive impairment		X		X	

a Self-management strategies include rest, good sleep hygiene, energy pacing, diet control, and distraction.

b Prior to physical exercise rehabilitation initiation, rule out conditions such as post-exertional malaise (PEM) and postural orthostatic tachycardia syndrome (POTS).

CVD, cardiovascular dysautonomia; SGB, stellate ganglion block; LDN, low-dose naltrexone.

### 5.1 Cardiopulmonary symptoms

Fatigue and dyspnea are two of the most common complaints in patients with long COVID (41, 42, 46–51). Fatigue screening may be achieved by utilizing validated tools such as the Fatigue Assessment Scale (FAS), Visual Analogue Fatigue Scale (VAFS), or Fatigue Severity Scale (FSS) (41, 43). Objective testing for fatigue may be performed using tests such as the 6-min walk test (6MWT) and the Timed Up and Go (TUG) Test (41). Dyspnea is associated with poor sleep, mood, life quality and strength particularly at 12 months post-COVID (52). Dyspnea and depressive symptoms at 3 months post-COVID are predictors of severity of dyspnea 12 months post-COVID and may be screened with standardized dyspnea and mood questionnaires (52). Assessment of dyspnea may include pulmonary function tests (PFTs), transthoracic echocardiogram (TTE), and the 6-min walk test (6MWT) (41). Patients with pre-existing cardiac history should have regular monitoring of troponin and inflammatory panels, electrocardiogram, chest x-ray, echocardiogram, holter-EKG and spirometry (18).

Cardiovascular dysautonomia symptoms include chest pain, palpitations, fatigue, and brain fog due to deficient function of the autonomic nervous system (ANS) (52). The most prevalent type is postural orthostatic tachycardia syndrome (POTS) (52, 53). POTS may be screened and assessed through utilization of orthostatic vital signs and tilt table testing (41, 53). Symptom management for patients with confirmed POTS may include compression stockings, hydration, and behavioral modification (41, 53). Pharmacological management may also be employed based on target symptom. For example, to reduce tachycardia and improve exercise or orthostatic intolerance, beta-blockers may be utilized (53).

Physical exercise rehabilitation has also been shown to provide improvement of fatigue but it is essential to rule out conditions such as post-exertional malaise (PEM) and POTS as exercise may exacerbate symptoms and be harmful (41, 48, 51). A randomized controlled trial (RCT) examined functional versus aerobic exercise telerehabilitation programs in combination with breathing techniques to improve long COVID symptoms and demonstrated both improved quality of life and stress symptoms (54). However, functional exercise exhibited more significant results improving fatigue and functional performance (54). Physical exercise rehabilitation should be performed in a clinical setting with direct supervision to ensure patient safety (51, 55).

Pulmonary rehabilitation has positive effects on dyspnea, physical function, quality of life, anxiety and depression, however not as significant on fatigue (56, 57). Face-to-face rehabilitation and telerehabilitation both demonstrate improved outcomes with face-to-face delivery faring slightly better in terms of improved quality of life (57).

### 5.2 Neuropsychiatric symptoms

The most common neuropsychiatric symptoms in long COVID include depression, anxiety, sleep disturbances, parosmia, and cognitive impairment (58, 59). “Brain fog” is a term used by patients to describe their cognitive impairment experience and may include any of the following: concentration difficulty, feelings of confusion, cognitive slowing, mental fuzziness, forgetfulness, word finding, mental fatigue (45, 59).

Neuropsychiatric screening may include formal psychological assessment, testing for autonomic dysfunction, and cognitive impairment screening with the MOCA test (26, 41). Sleep quality and mental health should also be assessed (41). Autonomic dysfunction may be screened with the Composite Autonomic Symptom Scale-31 (COMPASS 31) and diagnosis may be facilitated through evaluation of beat-to-beat blood pressure and heart rate variability (HRV) (26). Dysautonomia symptoms that are also components of long COVID include changes to smell and taste, headaches, and hypoxia (26).

Utilization of psychological aides such as cognitive behavioral therapy and antidepressants for mental health conditions associated with long COVID may provide benefit (26).

Parosmia is a sense of smell distortion and negatively impacts quality of life as a significant number of patients report associated weight loss, reductions in enjoyment of food, and depression (60). For persistent parosmia, management includes olfactory training, nasal corticosteroid sprays, and/ or vitamin A drops (41, 60).

Exercise based rehabilitation is also recommended to manage long COVID mental health and sleep-related problems once other conditions such as POTS and PEM have been ruled out (41, 48, 56).

### 5.3 Underutilized modalities

Long COVID symptoms contribute to social and economic hardship for individuals and their families highlighting the urgent need for interventions to provide relief (61). The following highlights two underutilized interventions with well-established safety profiles that may improve functionality and quality of life in patients suffering with long COVID.

#### 5.3.1 Stellate ganglion block

Overactivity in the sympathetic nervous system coupled with underactivity in the vagus nerve may contribute to the persistent inflammation found in long COVID (62). This persistent inflammation unsettles the sympathetic and parasympathetic nervous systems’ balance and likely contributes to the characteristic symptoms of long COVID (62). A safe and underutilized intervention to target the autonomic nervous system to potentially relieve long COVID symptoms is the stellate ganglion block (SGB). SGBs have been a treatment modality for nearly a century, particularly for several sympathetically mediated conditions such as complex regional pain syndrome (CRPS), postherpetic neuralgia, and refractory cardiac arrhythmias (32, 63). SGBs are considered an emergent modality in conditions such as anosmia, chronic fatigue syndrome, and anxiety and depression in PTSD (64).

SGBs are performed with imaging guidance while patients are in the supine position, head turned opposite from the procedure side, and with the head of the bed slightly elevated to reduce risk of adverse events such as pneumothorax or injury of adjacent structures (62, 64). This positioning also facilitates decompression of the subclavian vessels and reduces the distance of the stellate ganglion from the needle entry point (62).

Complications that may occur after SGB delivery include systemic or local adverse events. Systemic adverse events include the most common complaints of hoarseness and lightheadedness, followed by cough, dyspnea, migraine headaches, or ptosis (65). Local adverse events include hematoma formation, dural puncture, and local infection (two cases with patients on either concomitant oral or system steroids potentially increasing infection risk during the peri-procedure period) (65). While most complications are transient, these potential adverse events must be weighed against the patient’s personal symptom burden prior to SGB recommendation.

Parosmia is associated with poor quality of life and significant weight loss (60). In a study comparing interventions for parosmia, while SGBs had the lowest utilization in comparison to oral steroids and smell training, it had the highest reported percentage improvement among participants with maintained benefit (60). The classic protocol as designed by Hummel et al. for smell training, or olfactory training, involves patients sniffing four odors for at least 10 s, twice daily, for at least 3 months (66, 67).

A retrospective cohort study of 41 participants evaluated the use of SGBs for long COVID symptoms and initial symptoms included: fatigue (85%), brain fog (80%), post-exertional malaise (66%), mood changes (51%), taste or smell changes (44%), shortness of breath (41%), sleep problems (34%), tachycardia or palpitations (22%) (62). Reduction of at least one symptom was reported in 86% and relief of all presenting initial symptoms was reported in 61% of participants (62). Most patients reported symptom improvement within 15-min of SGB delivery while other symptoms that could not be evaluated immediately such as fatigue or brain fog, improvement was reported over 1–2 weeks from delivery (62). At 9-to-12-month follow-up, only 2 patients reported return of symptoms (62). Long term follow-up studies of long COVID patients after SGB are recommended to evaluate duration of benefit. Pearson et al. (62) urge for prompt consideration of SGBs to treat long COVID symptoms as diminished response has been observed in other chronic post-viral diseases such as Lyme disease or myalgic encephalopathy and is theorized to be attributed to time-critical neuroadaptive changes.

For patients suffering with long COVID, consider timely referral to interventional pain management for SGB consideration.

#### 5.3.2 Low-dose naltrexone

Another underutilized intervention with an established safety profile for a variety of conditions is the pharmacological agent naltrexone. Naltrexone is a non-selective opioid antagonist currently approved by the US Food and Drug Administration (FDA) for the treatment of alcohol and opioid dependence and is prescribed at 50–150 mg daily (68, 69). At doses below 5 mg, it is considered low-dose naltrexone (LDN), exhibits anti-inflammatory and analgesic properties, and has been used off-label to reduce severity of symptoms in conditions such as fibromyalgia, multiple sclerosis, complex regional pain syndrome, myalgic encephalomyelitis/chronic fatigue syndrome (ME/CFS), and Crohn’s disease (69–71). LDN is widely available with a prescription, also at a low cost, and is associated with minimal side effects (69). LDN has been associated with improvement of several clinical symptoms related to long COVID such as fatigue, poor sleep quality and pattern, brain fog, post-exertional malaise, headache, and demonstrated reduction of symptoms and improvement of functionality (70). In patients living with long COVID presenting with neuropsychiatric symptoms, fatigue, or exertional intolerance, consider utilization or adjunct therapy with LDN.

### 5.4 Prevention

There is general agreement across the literature stating the most effective way to prevent long COVID is to prevent COVID-19 infection with appeals for strong vaccination efforts (15, 18, 72). Vaccination may decrease prevalence of long COVID among US adults by almost 20.9% with at least two-dose vaccination associated with lower risk of persistent fatigue and pulmonary complaints (72, 73). Vaccination reduces the risk of long COVID (40, 41, 46, 74, 75). Several studies demonstrate protective effects of vaccination against long COVID (46, 76–79). Additionally, vaccination alleviates the severity of long COVID symptoms (26, 41, 80–82).

## 6 Conclusion

Long COVID is an emerging condition without formal consensus on its definition, diagnostic tools, or validated treatments. In a subset of patients, long COVID is likely a chronic disease with long-term disability which will contribute to rising healthcare utilization, and decreased workforce and productivity. There is an urgent need for more knowledge regarding long COVID identification, treatments, and outcomes to direct management guidelines particularly for primary care providers who serve as the gap to more specialized care for patients living with long COVID. Multidisciplinary rehabilitation is the most recommended course of care for patients with long COVID (2, 18). However, it is not recommended for patients with irreversible lung damage (70), ME/CFS (32), or POTS (18).

ME/CFS and long COVID have many overlapping symptoms and features making it difficult to distinguish one from another. Most notable differences include: changes to smell and taste, rash and loss of hair more likely in long COVID compared to ME/CFS (83). Higher inflammatory response reflected by stronger cytokine levels has been demonstrated in ME/CFS compared to long COVID however larger scale studies are needed to confirm (84). Employ caution with physical exercise rehabilitation recommendations as exercise may exacerbate symptoms and be detrimental for conditions such as PEM and POTS (41, 48, 51). In ME/CFS patients, NICE guidelines have recommendations for pacing methods for these patients as to not overexert themselves or aggravate their symptoms (32). Long COVID symptoms can be debilitating to its sufferers and impact not only patients but their families. In addition to multidisciplinary rehabilitation, consider early referral to interventional pain management for consideration of SGB or initiation of LDN to alleviate long COVID symptoms. Vaccination not only reduces the risk of and is protective against long COVID, but also alleviates the severity of long COVID symptoms (26, 40, 41, 46, 74–77, 80–82).

Primary care providers are at the forefront of care for their patients living with long COVID. With this up-to-date information, they will be able to identify the most common symptoms and presentation, validate patients’ experiences, provide care recommendations to improve quality of life and functionality, and coordinate future long-term supportive care.

## Author contributions

TD: Writing – review & editing, Writing – original draft, Visualization, Conceptualization. KB: Writing – review & editing.
